# L-isoleucine-supplemented Oral Rehydration Solution in the Treatment of Acute Diarrhoea in Children: A Randomized Controlled Trial

**DOI:** 10.3329/jhpn.v29i3.7864

**Published:** 2011-06

**Authors:** N.H. Alam, R. Raqib, H. Ashraf, F. Qadri, S. Ahmed, M. Zasloff, B. Agerberth, M.A. Salam, N. Gyr, R. Meier

**Affiliations:** ^1^ICDDR,B, GPO Box 128, Dhaka 1000, Bangladesh; ^2^Department of Research and Translational Science, Georgetown University, Washington, USA; ^3^Karolinska Institute, Stockholm, Sweden; ^4^University Hospital, Basel, Switzerland; ^5^University Hospital, Liestal, Switzerland

**Keywords:** Dehydration, Diarrhoea, Acute, Diarrhoea, Infantile, Double-blind method, Oral rehydration solutions, Randomized controlled trials, Bangladesh

## Abstract

Antimicrobial peptides represent an important component of the innate immune defenses of living organisms, including humans. They are broad-spectrum surface-acting agents secreted by the epithelial cells of the body in response to infection. Recently, L-isoleucine and its analogues have been found to induce antimicrobial peptides. The objectives of the study were to examine if addition of L-isoleucine to oral rehydration salts (ORS) solution would reduce stool output and/or duration of acute diarrhoea in children and induce antimicrobial peptides in intestine. This double-blind randomized controlled trial was conducted at the Dhaka Hospital of ICDDR,B. Fifty male children, aged 6-36 months, with acute diarrhoea and some dehydration, attending the hospital, were included in the study. Twenty-five children received L-isoleucine (2 g/L)-added ORS (study), and 25 received ORS without L-isoleucine (control). Stool weight, ORS intake, and duration of diarrhoea were the primary outcomes. There was a trend in reduction in mean±standard deviation (SD) daily stool output (g) of children in the L-isoleucine group from day 2 but it was significant on day 3 (388±261 vs 653±446; the difference between mean [95% confidence interval (CI) (-)265 (−509, −20); p=0.035]. Although the cumulative stool output from day 1 to day 3 reduced by 26% in the isoleucine group, it was not significant. Also, there was a trend in reduction in the mean±SD intake of ORS solution (mL) in the L-isoleucine group but it was significant only on day 1 (410±169 vs 564±301), the difference between mean (95% CI) (-)154 (-288, −18); p=0.04. The duration (hours) of diarrhoea was similar in both the groups. A gradual increase in stool concentrations of ß-defensin 2 and 3 was noted but they were not significantly different between the groups. L-isoleucine-supplemented ORS might be beneficial in reducing stool output and ORS intake in children with acute watery diarrhoea. A further study is warranted to substantiate the therapeutic effect of L-isoleucine.

## INTRODUCTION

Globally, diarrhoea still accounts for 1.6-2.5 million deaths each year; children in the developing world experience an average of three episodes of diarrhoea each year; and despite the impressive decline from 1980, it remains the second leading cause of childhood deaths (1). Most important elements of the management of acute diarrhoea include: (a) prevention of dehydration using home-made fluids or oral rehydration salts (ORS) solution; (b) correction of dehydration using ORS solution or intravenous fluid, as clinically indicated; (c) replacing ongoing stool losses followed by oral maintenance fluid; and d) continuation of usual food, including breastfeeding. Antimicrobial therapy is recommended in the management of diarrhoea caused by a few specific pathogens, such as severe cholera, shigellosis, invasive intestinal amoebiasis, and symptomatic giardiasis. The cost of therapy and, more importantly, the emergence of resistant pathogens are the major concerns for antimicrobial therapy for diarrhoea as it is for other bacterial infections. A major limitation of ORS—old formulation (Na-90 mmol/L, Cl-80 mmol/L, K-20 mmol/L, citrate-10 mmol/L, glucose anhydrous-111 mmol/L, total osmolarity 311 mosmol) (including current ORS: Na-75 mmol/L, Cl-65 mmol/L, K-20 mmol/L, citrate-10 mmol/L, glucose, anhydrous-75 mmol/L, total osmolarity 245-mosmol/L) is that it neither reduces the duration of diarrhoea nor its severity, and efforts are, thus, continuing to overcome these limitations by developing newer formulations of ORS (2-7).

Antimicrobial peptides represent an important component of the innate immune defenses of organisms ranging from plants to insects to humans. They are membrane-active agents that kill microbes by various mechanisms (8,9). Most have a broad spectrum of activities that include bacteria, viruses, and fungi. In humans, two major classes of antimicrobial peptides—defensins and cathelicidins (LL-37)—have been described. The human defensins are of two types: α and ß-defensins. α-defensins are mostly present in neutrophil granules necessary for non-oxidative killing of phagocytosed microbes and those in the Paneth cells in the crypts of Leiberkuhn in the small intestine (10). ß-defensins, present in virtually all epithelial surfaces, including those of the skin, airways, gut, and urogenital tracts (11-14), contribute to the antimicrobial barrier at the epithelial surface. Impairment of defensin functions increases susceptibility to infection of the airway in cystic fibrosis (15) and enhances *Salmonella* infection in the intestinal tract of mouse (16). Many antimicrobial peptides expressed on epithelial surfaces are induced in the settings of infection or injury (8,9). Pattern-recognition receptors (17) likely play a critical role in this process as has been shown in CD-14-mediated induction of ß-defensins by bacterial lipopolysaccharide (18). Whole, heat-killed bacteria and fungi induce human ß-defensins (HBD)-2 in human keratinocytes but the molecular basis of this response is not understood (19). Inflammatory cytokines also induce epithelial ß-defensins (20-22). Pharmacological induction of antimicrobial peptides at epithelial barriers could have therapeutic utility (23,24). Several groups have recently reported the discovery of substances with low molecular weight that induce epithelial antimicrobial peptides in cell-based assays (23,24). L-isoleucine and its analogues are highly-specific ß-defensin inducers in epithelial cells (24,25). Butyrate has also been shown to induce antimicrobial peptides in the colonic epithelial cells and has been shown to exhibit therapeutic benefit in a rabbit model of shigellosis (23); however, it cannot be administered orally as such for its foul smell.

In this pilot study, we have evaluated orally-administered L-isoleucine in the treatment of acute infectious diarrhoea in infants and young children. The study was driven by the hypothesis that L-isoleucine enhances the secretion of antimicrobial peptides in the intestinal epithelium and might favourably impact the resolution of disease. Therefore, we undertook a study to determine whether the use of L-isoleucine-supplemented ORS solution would enhance clinical recovery of children with acute watery diarrhoea and whether the treatment could be correlated with the presence of increased concentrations of antimicrobial peptides in stool.

## MATERIALS AND METHODS

### Study design

In this pilot randomized, double-blind controlled clinical trial, we enrolled 50 infants and young children—25 in the study group and 25 in the control group. The study patients were selected from among male children, aged 6-36 months, who attended the Dhaka Hospital of ICDDR,B during July 2007–June 2008, with a history of acute watery diarrhoea of less than 48 hours’ duration with some dehydration. Those with cholera (stool dark-field microscopy positive for *Vibrio cholerae*), dysentery (presence of visible blood in stool), severe dehydration, severe malnutrition (weight-for-age <60% or weight-for-length <70% of the National Center for Health Statistics median or with nutritional oedema), and history of taking antimicrobial or antidiarrhoeal drugs for the current illness were not eligible.

### Patient management and treatment schedule

Children fulfilling the eligibility criteria were admitted to the study ward of the Dhaka Hospital of ICDDR,B and weighed. Their detailed medical history was obtained. A thorough physical examination, including assessment of dehydration, was done. All findings were recorded in pre-designed Case Report Forms (CRFs). The status of dehydration was assessed following the guidelines of the World Health Organization (WHO) (26). Children were then randomized to receive either: (a) WHO and United Nations Children's Fund-recommended hypo-osmolar glucose-ORS (Na-75 mmol/L, Cl-65 mmol/L, K-20 mmol/L, citrate-10 mmol/L, and glucose-75 mmol/L) plus L-isoleucine-2 g/L-calculated osmolality–252 mosmol/L (study group), or (b) the same ORS without L-isoleucine (control group). The dose of L-isoleucine was arbitrarily selected. A trained staff member of ICDDR,B, not involved in the study, prepared the randomization list. The name of intervention was indicated on a paper, kept inside the envelope. The sealed envelopes were supplied to the Dhaka Hospital pharmacists, not involved in the study in any way, to prepare the solutions, which were identical in appearance and similar in taste. They labelled the bottles with the assigned random number, patient's name, and hospital registration number and handed these over to the study staff for making ready to drink. L-Isoleucine is a chemically and physically-stable and neutral amino acid, tasteless, and colourless at the concentration used in ORS (http://www.ajiaminoacids.com/default.aspx). After 12 hours, unused ORS was measured and discarded, and a fresh bottle of ORS was prepared for continuation of therapy, if required. Oral rehydration therapy (ORT) was continued until resolution of diarrhoea but up to a maximum of five days. All children received zinc tablet. Infants, aged 6-12 months, received 10 mg daily, and children, aged over 12 months, received 20 mg daily for 10 days. Children on breastmilk was allowed to receive breastmilk, and supplementary diet prepared from cows’ milk, rice-powder, and sugar (*milk suji*) delivering 78 kcal/100 g was administered to those aged over six months (10 mL/kg every two hours for at least 10 feeds) following the standard practice of the hospital.

### Laboratory investigations

Routine laboratory investigations included microscopic examination of fresh stool specimens for enumeration of leucocytes and red blood cells and identification of parasites, such as *Giardia lamblia*, *Entamoeba histolytica*, and *Cryptosporidium*, and for culture to isolate and identify *Salmomella, Shigella, V. cholerae,* and *Campylobacter jejuni* by standard methods and for enzyme-linked immunosorbent assay (ELISA) to demonstrate the presence of rotavirus.

### Clinical assessments

The children were placed on a ‘cholera-cot’, and a paediatric urine-collector (PUC bag) was used for collecting urine separately. Stool-weight, supplemented food, and body-weight were measured with an electronic scale (Sartorius, Göttingen, Germany) with a precision of 1 g. ORS, plain water, and urine were measured with a calibrated cylinder. Measurements of all intakes (ORS, food, and water) and output (stool, urine, and vomit) were summarized for every six-hour period of the study until discharge. Body-weight was also measured every six hours until the children were discharged. Physical examinations were performed in the morning and in the afternoon. Resolution of diarrhoea was defined as the appearance of soft or formed stool or no stool for 12 hours. The participating patients were discharged after the resolution of diarrhoea but not before 72 hours. Therapeutic success was defined as cessation of diarrhoea within five days of the study intervention. The duration of diarrhoea was calculated from the time of randomization to the last watery stool.

### Assessments of antimicrobial peptides in stool

Stool specimen (approximately 3-5 g) from each patient was collected at the end of each 24-hour study period, mixed with a buffer containing 60% acetonitrile and 10% trifluoroacetic acid in water in a ratio of 1:10, mixed vigorously, kept overnight at 4 °C in a shaking incubator, and centrifuged at 13,000 rpm for 30 minutes. Supernatant was filtered with 0.2 µm filter, lyophilized, and stored at −20 °C until used. Before using in ELISA, lyophilized stool was dissolved in phosphate buffered saline (PBS) in one-third of the starting volume (27). Cathelicidin LL-37 was measured in stool by ELISA as described earlier (28). ELISA plates (Maxisorp by Nunc, Naperville, IL, USA) were coated with monoclonal LL-37 antibody (5 µg/mL in coating buffer, pH=9.6) at 4 °C overnight. After washing, 0.1% gelatin in PBS was added to block unspecific binding for one hour at room temperature. Samples or standard LL-37 peptide, in a serial dilution (0.1-1,000 ng/mL in PBS), were added in duplicate and incubated at 4 °C overnight, followed by incubation with biotinylated polyclonal LL-37 antibody (20 µg/mL; 0.1% gelatin in PBS) at room temperature for two hours and incubation with streptavidin-AP (1:2000; 0.1% gelatin in PBS) at room temperature for two hours. Between the incubations, plates were washed three times with PBS (0.05% Tween). Finally, plate was developed with p-nitrophenylphosphate (l mg/mL) in diethanolamin buffer (pH=9.8), and the absorbance was measured at 405 nm after 30–60 minutes. ß-defensin (HBD) 1 and 3 were measured in stool extracts using ELISA kits (Phoenix Pharmaceuticals, Inc., Burlingame, CA) following the instructions of the manufacturer.

### Analysis of data

Data were analyzed using the SPSS PC+ software (version 10) (University of Chicago, USA). Continuous variables were compared between the groups with Student's *t*-test, and χ^2^ was used for comparing the dichotomous variables. A survival plot was constructed and compared with log rank rest. Data were not normally distributed, such as antimicrobial peptides (HBD-2 and HBD-3) transformed into square root, and analysis was performed with two-way analysis of variance (ANOVA).

### Ethical issues

Written, informed consent was obtained from parents or the legal guardians of each of the study children. The Research Review Committee and the Ethical Review Committee of ICDDR,B approved the study protocol.

## RESULTS

### Effects of treatment on clinical response

Since this is an exploratory pilot study, we studied 50 patients—25 received L-isoleucine-supplemented ORS (L-isoleucine group), and 25 received ORS without L-isoleucine (control group). Baseline clinical characteristics, such as age, body-weight, weight-for-length (%) of NCHS median, weight-for-age (%) of NCHS median, duration of diarrhoea before admission, duration of vomiting before admission, dehydration status, breastfeeding status, etc., were comparable between the groups (Table 1). The major outcome variables were stool output, ORS intake, and duration of diarrhoea. With respect to stool output, we noted a trend in reduced output in children receiving ORS supplemented with L-isoleucine on day 1, which continued on day 2 and became statistically significant (p=0.03) on day 3 (Table 2). Also, we observed a trend in reductions in the volume of ORS intake in the L-isoleucine group compared to the control group, although statistical significance (p=0.04) was only observed on day 1. These findings may be due to the small sample-size. We did not observe a reduction in the duration of diarrhoea in children receiving L-isoleucine-supplemented ORS (Table 2). Kaplan Meier survival plot (Fig.) for duration of diarrhoea also failed to detect any significant difference between the groups. There were no adverse effects associated with the intake of L-isoleucine-supplemented ORS.

**Table 1.** Baseline characteristics of study children

Variable	Isoleucine-added ORS group (n=25)	Control ORS group (n=25)	p value
Age (months)	10.4±3	12.0±4.5	0.12
Body-weight (kg)	7.7±1.0	8.0±1.14	0.36
Weight-for-age (%)	81.0±8.2	79.5±10.6	0.59
Weight-for-length (%)	87.4±8.3	88.1±9.0	0.76
Duration (hours) of diarrhoea before admission	28.0±11.5	27.9±13.5	0.95
Number of stools in the last 24 hours	12±4	11±4	0.88
Duration (hours) of vomiting before admission	26.9±13.0	24.6±15	0.56
Number of vomits in the last 24 hour	7.5±3	7.5±4	0.98
Breastfeeding (yes/no)	24/2	22/3	0.67
Dehydration status (none/some)	0/26	0/25	
Fever before admission (yes/no)	14/12	10/15	0.57
Stool pathogens isolated			
Enterotoxigenic *Eschericia coli*	3	3	0.72
Rotavirus	18	17	

Figures represent mean±SD or numbers.

ORS=Oral rehydration solutions;

SD=Standard deviation

**Table 2.** Comparison of outcome variables

Variable	Isoleucine-added ORS group (n=25)	Control ORS group (n=25)	Difference between mean (95% CI)	p value
Stool output (g)—day 1	560±240	563±409	-3.0 (-189,183)	0.94
Stool output (g)—day 2	407±284	515±316	-108 (-285, 71)	0.23
Stool output (g)—day 3	388±261	653±446	-265 (-509, −20)	0.035
ORS intake (mL)—day 1	410±169	564±301	-154 (-288, −18)	0.04
ORS intake (mL)—day 2	330±245	401±226	-71 (-212, 70)	0.23
ORS intake (mL)—day 3	312±233	454±260	-142 (-312, 28)	0.09
Duration (hours) of diarrhoea	74±38	75±42	-1 (-17, 25)	0.96

Figures represent mean±SD.

CI=Confidence interval;

ORS=Oral rehydration solution;

SD=Standard deviation

**Fig.**
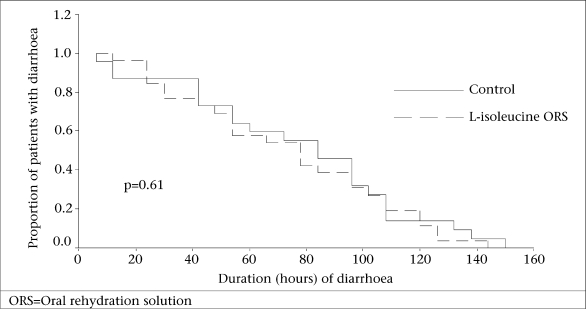
Kaplan-Meir survival curve for duration of diarrhoea

### Effects of treatment on ß-defensin levels in stool

We measured the stool concentrations of four antimicrobial peptides (LL-37, HBD-1, HBD-2, and HBD-3) expressed in the lumen of small and large intestines. Measurements were made at the start of treatment (day 0), after two days of treatment (day 2), and after diarrhoea had resolved (day 7). Stool sample for day 7 was collected as ambulatory. LL-37 peptide measured in stool samples was barely detectable in most patients. Two-way ANOVA did not show any difference in stool LL-37 levels between days (p=0.47) or treatment groups (p=0.10) (data not shown). Concentrations of HBD-1 in stool were barely detectable and were similar in both the groups of patients. Concentrations of HBD-2 in stool increased significantly in both the groups between days (p=0.0001) but no difference between treatment groups could be observed (p=0.5). Concentrations of HBD-3 in stool, however, increased significantly (p=0.017) in the intervention group from day 2 to day 7 but showed no consistent pattern in the control group. Two-way ANOVA showed a significant difference (p=0.02) between days but not between treatment groups (p=0.51). However, there were interactions that showed a trend for significance (p=0.08) (Table 3). Within day 7, concentration of HBD-3 was significantly higher (p=0.027) in the isoleucine group compared to the control group.

**Table 3.** Concentration of beta-defensin-3 (HBD-3) and beta-defensin-2 (HBD-2) in stool from patients treated with or without L-isoleucine in oral rehydration solution

Antimicrobial peptide	Time	Control	Supplemented	Two-way ANOVA	p value
HBD-3	Day 0	238±12	37±0	Time	0.023
	Day 2	37±0	62±1	Supplement	0.518
	Day 7	153±6	51±24	Interaction	0.080
HBD-2	Day 0	9±5	44±18	Time	0.001
	Day 2	39±9	63±11	Supplement	0.56
	Day 7	123±41	132±28	Interaction	0.62

Data expressed as mean±standard deviation.

Concentration of beta-defensin-3 and beta-defensin-2 in stool is expressed in pg/g stool.

ANOVA=Analysis of variance

## DISCUSSION

The results of this pilot study demonstrated that L-isoleucine-supplemented ORS showed some beneficial effects in terms of reducing stool output and ORS intake in children with non-cholera acute watery diarrhoea compared to the control group, albeit without the anticipated shortening of its duration. To our knowledge, this is the first study that evaluated the effect of L-isoleucine-added ORS on stool output and recovery in patients with acute non-cholera diarrhoea. Some studies have, however, evaluated the effect of other amino acids, e.g. alanine, glycine, and glutamine, added to ORS in the treatment of infectious diarrhoeas (2-5). Some of these studies have shown beneficial effects in terms of reducing stool output while these ORSs were used in cholera patients but those amino acids-supplemented ORSs had no significant impact when used for the treatment of acute non-cholera diarrhoea in young children. The amino acids, such as alanine, glycine, and glutamine, were thought to enhance absorption of sodium and water from the small intestine when added to ORS. Whether L-isoleucine can also enhance absorption of sodium and water in our study patients in the small intestine and, in so doing, reduce stool output, was not investigated in the present study. Further studies may establish the effect of antimicrobial peptides by any agents blocking their effect.

We postulated that supplementation with L-isoleucine would induce antimicrobial peptides in the intestine that would have activities against enteric bacteria and viruses. Of the antimicrobial peptides measured in the present study, HBD-3 exhibited an increase in stool concentrations over the days in the L-isoleucine-supplemented group, although there was a non-significant difference between the two groups. We did not see any significant difference in the concentrations of LL-37, HBD-1, and HBD-2 between the two groups.

HBD-3 is inducible and is expressed by small intestinal and colonic epithelial cells, particularly in the crypt regions, in addition to their direct antimicrobial activities; ß-defensins are chemotactic for memory T cells and dendritic cells, suggesting that they play an important role in the integration of the innate and acquired immune responses (16). Upregulation of HBD-3 by isoleucine may additionally play a role in immune modulation in acute watery diarrhoea. Isoleucine is essential for normal growth and differentiation of enterocytes and keratinocytes, and treatment with isoleucine may, thus, support epithelial cell regeneration during diarrhoea.

### Limitations

Our study has several limitations. The bacterial and viral load in stool was not determined to show bacterial/viral clearance from stool after treatment. The epithelial expression of the antimicrobial peptides in the small intestine was not assessed; release of peptides in stool is a total output from the total gut. We did not assess whether the released HBD-2 or HBD-3 peptides were in active form or pro-form. The study being an exploratory pilot one had a small sample-size that might have been inadequately powered to distinguish several parameters measured between the groups, for which only a trend was observed. We selected a single low-dose (arbitrarily) of isoleucine on the basis of safety for this first study and did not explore the effects of higher doses.

### Conclusions

This preliminary study opens a new field of research—the clinical study of chemically simple, inexpensive, and safe substance to induce endogenous antimicrobial peptides to achieve a therapeutic benefit in an infectious disease. We anticipate that such agents should they prove effective would have applications in the prevention and treatment of infectious diseases and could reduce the use of conventional antibiotics.

#### What is already known on this topic

Antimicrobial peptides secreted from the epithelial surfaces of all living animals and plants represent an important component of the innate immune defenses of organisms. They are membrane-active agents that kill microbes by various mechanisms. Recently, L-isoleucine and its analogues have been reported to induce antimicrobial peptides in epithelial surfaces of the intestine. However, its therapeutic potential has not been evaluated.

#### What this study adds

So far, this is the first study that has evaluated the therapeutic effect of L-isoleucine added to ORS in the treatment of acute diarrhoea in children. Although this is the pilot and exploratory study, the preliminary results showed some beneficial effects in terms of reduction of stool output and ORS intake. Although induction of antimicrobial peptides in response to L-isoleucine was not significantly increased compared to the control, there were indications. Thus, we recommend more mechanistic studies to establish the therapeutic and antimicrobial-inducing effect of L-isoleucine.

## ACKNOWLEDGEMENTS

The study was funded by the Gastroenterology Science Foundation, University of Basel, Switzerland. ICDDR,B acknowledges with gratitude the commitment of the Gastroenterology Science Foundation, University of Basel, to its research efforts.
